# Nargenicin A1 attenuates lipopolysaccharide-induced inflammatory and oxidative response by blocking the NF-κB signaling pathway

**DOI:** 10.17179/excli2021-3506

**Published:** 2021-05-28

**Authors:** Da Hye Kwon, Gi-Young Kim, Hee-Jae Cha, Suhkmann Kim, Heui-Soo Kim, Hye-Jin Hwang, Yung Hyun Choi

**Affiliations:** 1 Anti‐Aging Research Center, Dong‐eui University, Busan, Republic of Korea; 2 Department of Biochemistry, Dong‐eui University College of Korean Medicine, Busan, Republic of Korea; 3 Department of Marine Life Science, School of Marine Biomedical Sciences, Jeju National University, Jeju, Republic of Korea; 4 Department of Parasitology and Genetics, College of Medicine, Kosin University, Busan, Republic of Korea; 5 Department of Chemistry, College of Natural Sciences, Pusan National University, Busan, Republic of Korea; 6 Department of Biological Sciences, College of Natural Sciences, Pusan National University, Busan, Republic of Korea; 7 Department of Food and Nutrition, College of Nursing, Healthcare Sciences & Human Ecology, Dong-eui University, Busan, Republic of Korea

**Keywords:** nargenicin A1, inflammation, ROS, NF-kappaB

## Abstract

Inflammation caused by the excessive production of pro-inflammatory mediators and cytokines in abnormally activated macrophages promotes the initiation and progression of many diseases along with oxidative stress. Previous studies have suggested that nargenicin A1, an antibacterial macrolide isolated from *Nocardia *sp. may be a potential treatment for inflammatory responses and oxidative stress, but the detailed mechanisms are still not well studied. In this study, we investigated the inhibitory effect of nargenicin A1 on inflammatory and oxidative stress in lipopolysaccharide (LPS)-stimulated RAW 264.7 macrophages and zebrafish (*Danio rerio*) models. Our results indicated that nargenicin A1 treatment significantly inhibited LPS-induced release of pro-inflammatory mediators including nitric oxide (NO) and prostaglandin E_2_, which was associated with decreased inducible NO synthase and cyclooxygenase-2 expression. In addition, nargenicin A1 attenuated the LPS-induced expression of pro-inflammatory cytokines, such as tumor necrosis factor (TNF)-α, interleukin (IL)-1β, IL-6, and monocyte chemotactic protein-1, reducing their extracellular secretion. Nargenicin A1 also suppressed LPS-induced generation of reactive oxygen species. Moreover, nargenicin A1 abolished the LPS-mediated nuclear translocation of nuclear factor-kappa B (NF-κB) and the degradation of inhibitor IκB-α, indicating that nargenicin A1 exhibited anti-inflammatory effects by inhibiting the NF-κB signaling pathway. Furthermore, nargenicin A1 showed strong protective effects against NO and ROS production in LPS-injected zebrafish larvae. In conclusion, our findings suggest that nargenicin A1 ameliorates LPS-induced anti-inflammatory and antioxidant effects by downregulating the NF-κB signaling pathway, and that nargenicin A1 can be a potential functional agent to prevent inflammatory- and oxidative-mediated damage.

## Introduction

Inflammation is a type of protective immune responses against various toxic or irritating conditions, such as bacterial or viral infections, harmful stimuli, and cellular damage (Aksentijevich et al., 2020[1]; Medzhitov and Horng, 2009[24]). A properly controlled inflammatory response helps the body resist the insults, but abnormal or excessive inflammation causes hyperactivity in the body and is harmful. Excessive inflammatory reactions have been considered a major cause of diseases such as rheumatoid arthritis, diabetes, pulmonary fibrosis, degenerative diseases, atherosclerosis, chronic hepatitis, and various cancers (Semb et al., 2020[33]; Aksentijevich et al., 2020[1]; Liu et al., 2018[22]). Among the cells involved in the regulation of the immune system, macrophages play an important role in the innate immune defense system. Lipopolysaccharide (LPS) is a major component of Gram-negative bacterial cell walls and is widely used in a variety of assays to study the interference of inflammatory pathways in macrophage model. In LPS-stimulated macrophages, the production and release of pro-inflammatory mediators and cytokines is increased by activation of the toll-like receptor (TLR) 4-mediated nuclear factor-kappaB (NF-κB) signaling pathway (Hernandez et al., 2019[16]; Doyle and O'Neill, 2006[11]). Nitric oxide (NO) and prostaglandin E_2_ (PGE_2_) are representative pro-inflammatory mediators, and cytokines such as tumor necrosis factor (TNF)-α, interleukin (IL)-1β, IL-6, and monocyte chemotactic protein-1 (MCP-1) facilitate the inflammatory response (Aleem and Tohid, 2018[2]; Wyns et al., 2015[38]). Moreover, the expression of inducible NO synthase (iNOS) and cyclooxygenase-2 (COX-2), which are involved in the production of NO and PGE_2_, respectively, is positively correlated with the expression of pro-inflammatory cytokines (Soufli et al., 2016[35]; Förstermann and Sessa, 2012[14]). Their inadequate activation or abnormal upregulation is common in most inflammatory diseases (Saini and Singh, 2019[31]; Yao and Narumiya, 2019[39]; Soufli et al., 2016[35]).

On the one hand, reactive oxygen species (ROS) and related species are essential for proper physiological function and serve as important signaling molecules that are closely related to host defense responses. However, oxidative stress, characterized by the excessive production of ROS, contributes to the initiation and progression of the inflammatory response and diseases (Liu et al., 2018[22]; Bjørn and Hasselbalch, 2015[4]). Upon the LPS stimulation of macrophages, the production of ROS is also increased, contributing to the manifestation of inflammation, and overproduced inflammatory factors may promote excessive ROS production (Liu et al., 2018[22]; Mills and O'Neill, 2016[25]). Recently, zebrafish (*Danio rerio*), which have great advantages as an *in vivo* animal model, is widely used as a powerful vertebrate animal model for the study of human diseases (Bailone et al., 2020[3]; de Abreu et al., 2019[8]). In particular, since LPS-stimulated zebrafish exhibit inflammatory and oxidative reactions similar to those of mammals, and is recognized as an optimal *in vivo *model for evaluating anti-inflammatory and antioxidant efficacy of various drugs (Rodríguez-Ruiz et al., 2020[30]; Forn-Cuní et al., 2019[13]). Currently, nonsteroidal anti-inflammatory drugs are widely prescribed to relieve inflammatory symptoms and relieve oxidative stress, but various side effects have been reported with long-term use (Ferrer et al., 2019[12]; Utzeri and Usai, 2017[36]; Ghosh et al., 2015[15]). Therefore, there is an urgent need for research on reliable and effective alternatives for the prevention and treatment of various diseases.

Nargenicin A1 is a type of antimicrobial macrolide derived from the species *Nocardia*, an actinomycetes known as a source of biomolecules with various pharmacological activities (Dhakal et al., 2017[9], 2019[10]). In particular, this compound has been reported to have an effective anti-bacterial action against a variety of Gram-positive bacteria, including methicillin-resistant *Staphylococcus aureus* (Sohng et al., 2008[34]; Cane and Yang, 1985[5]; Magerlein and Reid, 1982[23]). Nargenicin A1 was also shown to promote leukemia cell differentiation while inhibiting leukemia cell proliferation, indicating that it has potential for the treatment of neoplastic diseases (Kim et al., 2009[20]). In addition, it has been suggested that nargenicin A1 can be applied to the treatment of inflammatory neurodegenerative diseases based on the results of inhibiting the inflammatory response by LPS in microglia (Yoo et al., 2009[40]). Recently, we have reported that nargenicin A1 could protect tacrolimus-induced oxidative stress-mediated DNA damage and apoptosis in hirame natural embryonic cells (Park et al., 2019[26]). However, the precise mechanisms for the inhibitory effects by nargenicin A1 on LPS-stimulated inflammatory and oxidative responses have not been well studied. Therefore, in this study, we investigated the effects of nargenicin A1 on LPS-stimulated RAW 264.7 macrophages and to elucidate the mechanisms involved. We also demonstrated the anti-inflammatory and antioxidant potential of nargenicin A1 in zebrafish.

## Materials and Methods

### Cell culture 

The RAW 264.7 cell line (ATCC^®^ TIB-71^™^), derived from murine macrophages, was purchased from the American Type Culture Collection (Manassas, VA, USA). The cells were maintained in humidified air at 37 °C, and 5 % CO_2_ in Dulbecco's modified Eagle's medium (DMEM, WelGENE Inc., Daegu, Republic of Korea) containing 100 U/ml penicillin and streptomycin, and 10 % fetal bovine serum (WelGENE Inc.). Nargenicin A1 and LPS were purchased from Sigma-Aldrich Chemical Co. (St. Louis, MO, USA). They were dissolved in dimethyl sulfoxide (DMSO, Sigma-Aldrich Chemical Co.) to make the stock solutions. Each stock solution (Nargenicin A1 1 mM, LPS 100 mg/ml) was appropriately diluted in the complete culture medium and used to treat RAW 264.7 macrophages.

### Cell viability assay 

The cytotoxicity of nargenicin A1 against RAW 264.7 macrophages in the presence or absence of LPS was determined using the 3-(4,5-dimethylthiazol-2-yl)-2,5-diphenyltetrazolium bromide (MTT) reduction assay as previously described (Choi, 2021[7]). In brief, the cells were treated with various concentrations of nargenicin A1 alone or pre-treated with the indicated concentrations of nargenicin A1 for 1 h before 100 ng/ml LPS treatment for 24 h. After that, the medium was removed, and MTT solution (0.5 mg/ml, Sigma-Aldrich Chemical Co.) was dispensed into each well and reacted at 37 °C for 3 h. Then, the supernatant was removed and DMSO was added for 10 min to dissolve the blue formazan crystals. The optical density was measured by an enzyme-linked immunosorbent assay (ELISA) plate reader (Dynatech Laboratories, Chantilly, VA, USA) set at 540 nm.

### Measurement of NO, PGE_2_, and cytokines 

RAW 264.7 cells were treated with different concentrations of nargenicin A1 for 1 h and then stimulated with LPS for 24 h. The NO level in the culture supernatant was estimated by the amount of nitrite measured using the Griess reagent (Sigma-Aldrich Chemical Co.) as previously described (Chae, 2020[6]). Briefly, 100 μL of the cell-conditioned medium was mixed with an equal volume of Griess reagent for 10 min. The absorbance was measured at 540 nm using an ELISA reader and calculated by comparison to a sodium nitrite (NaNO_2_) standard curve. To investigate the PGE_2_ and cytokine levels, the culture supernatants were collected and assayed using commercially available ELISA kits (R&D Systems Inc., Minneapolis, MN, USA) according to the instructions from the manufacturer. The absorbance was measured at a wavelength of 450 nm using an ELISA reader as previously described (Chae, 2020[6]). 

### RNA isolation and reverse transcription-polymerase chain reaction (RT-PCR) assay

Total RNA was extracted from the cells using TRIzol reagent (Invitrogen Life Technologies, Carlsbad, CA, USA), following the manufacturer's instructions, and quantified. The isolated total RNA (1 μg) was used to synthesize cDNA using AccuPower^®^ RT PreMix (Bioneer, Daejeon, Republic of Korea) according to the manufacturer's instructions. The cDNA generated at room temperature (RT) was amplified using the One-Step RT-PCR PreMix Kit with selected primers (iNtRON Biotechnology Inc., Seongnam, Republic of Korea). The amplified DNA products were electrophoresed on 1.5 % agarose gels and visualized after ethidium bromide (EtBr, Sigma-Aldrich Chemical Co.) staining as previously described (Kim et al., 2009[20]). Densitometric analysis of the bands was performed using the ImageJ® software (v1.48, NIH, Bethesda, MD, USA).

### Protein isolation and Western blot analysis

To extract whole proteins, the cells were washed with cold phosphate-buffered saline (PBS) and lysed with lysis buffer as previously described (Park et al., 2020[27]). In parallel, nuclear extraction reagents purchased from Pierce (Rockford, IL, USA) were used to isolate proteins from the nucleus and cytoplasm according to the manufacturer's protocol. Equal amounts of protein were separated by sodium dodecyl sulfate-polyacrylamide gel electrophoresis. Proteins in the gel were subsequently transferred to polyvinylidene difluoride (PVDF) membranes (Schleicher and Schuell GmbH, Keene, NH, USA). After blocking with non-fat dry milk solution (5 %) at RT for 1 h, the protein-transferred membranes were reacted with primary antibodies obtained from Santa Cruz Biotechnology, Inc. (Santa Cruz, CA, USA) and Cell Signaling Technology (Beverly, MA, USA) overnight at 4 °C. The membranes were washed three times for 5 min with Tris-buffered saline (0.1 % Tween-20) and then incubated with goat anti-rabbit IgG-horseradish-peroxidase (HRP) and goat anti-mouse IgG-HRP secondary antibodies (Santa Cruz Biotechnology, Inc.) for 2 h at RT. The membrane was reacted with an enhanced chemiluminescent solution purchased from Amersham Corp. (Arlington Heights, IL, USA) and then exposed to X-ray film to visualize the corresponding proteins. Densitometric analysis of the bands was performed using the ImageJ® software.

### Immunofluorescence for NF-κB

RAW 264.7 cells were seeded into 4-well cell culture slides and stabilized for 24 h. The cells were pre-treated with 10 µM nargenicin A1 for 1 h and then treated with or without 100 ng/ml LPS for 1 h. After treatment, the cells were fixed with ice-cold methanol for 10 min and washed with PBS. Subsequently, the cells were blocked using 5 % bovine serum albumin (BSA, Sigma-Aldrich Chemical Co.) with PBS-T (PBS containing 0.1 % Triton X) for 1 h and then incubated with anti-NF-κB (1:100 in 2.5 % BSA in PBS-T) at 4 °C overnight. The cells were washed with PBS-T and incubated with the secondary antibody (goat anti-rabbit IgG cross-absorbed secondary antibody conjugated to Alexa Fluor 594, Thermo Fisher Scientific, Waltham, MA, USA) for 1 h. After washing with PBS, the cells were counterstained with 4',6-diamidino-2-phenylindole (DAPI, Sigma-Aldrich Chemical Co.) for 20 min. Cell fluorescence was observed using a fluorescence microscope (Carl Zeiss, Oberkochen, Germany) at Core-Facility Center for Tissue Regeneration (Dong-eui University, Busan, Republic of Korea).

### Measurement of ROS levels

ROS was measured using 5,6-carboxy-2',7'-dichlorofluorescein diacetate (DCF-DA, Sigma-Aldrich Chemical Co.). Briefly, RAW 264.7 macrophages were pre-treated with various concentrations of nargenicin A1 for 1 h and then incubated for 1 h in the absence or presence of 100 ng/ml LPS. The cells were stained with 10 μM DCF-DA for 15 min in the dark at 37 °C. The cells were then washed with PBS and immediately analyzed by flow cytometry (BD Biosciences, San Jose, CA, USA) as previously described (Hwangbo et al., 2020[18]). To compare the degree of ROS generation through fluorescence microscopic observation, the cells were stained with DCF-DA for 15 min at 37 °C and then fixed with paraformaldehyde solution (4 %, pH 7.4) for 20 min. The cells were washed with PBS and analyzed for ROS fluorescence intensity using a fluorescence microscope.

### Zebrafish maintenance and LPS microinjection

AB strain zebrafish were provided by Dr. CH Kang (Nakdong National Institute of Biological Resources, Sangju, Republic of Korea) and maintained at 28.5 °C with a 14/10 h light/dark cycle according to the standard guidelines of the Animal Care and Use Committee of Jeju National University (Approval No.: 2019-0053, Jeju, Republic of Korea). Fertilized embryos were collected after natural spawning as previously described (Jeong et al., 2018[19]) and cultured in 2 mg/L methylene blue containing E3 embryo media at 28.5 °C. Three days post-fertilized (dpf) zebrafish larvae were anesthetized using 0.04 % tricaine (Sigma-Aldrich Chemical Co.) and LPS (0.5 mg/mL, 2 nL in each larva) was microinjected into the yolk using a Drummond NANOJECT III injector (Drummond Scientific, Broomall, PA, USA). The negative control group was injected with PBS. The larvae were washed three times after LPS microinjection and immediately placed in E3 media containing the indicated concentrations of nargenicin A1. Each group of larvae was cultured at 28.5 ℃ for 24 h. 

### NO and ROS staining in zebrafish larvae

The production of NO and ROS in zebrafish larvae was visualized using 4-amino-5-methylamino-2'7'-difluorofluorescein diacetate (DAF-FM-DA, Sigma-Aldrich Chemical Co.) and DCF-DA, respectively, 24 h after chemical treatment as previously described (Jeong et al., 2018[19]). In brief, zebrafish embryos (4 dpf) were transferred to 24-well plates and incubated with 5 µM DAF-FM-DA and 20 µM DCF-DA for 30 min and visualized using the CELENA^®^ S Digital Imaging System (Logos Biosystems, Anyang, Gyeonggido, Republic of Korea). Fluorescence intensities were calculated using ImageJ software (Wayne Rasband, National Institute of Health, Bethesda, MD, USA) and expressed as a percentage compared to the untreated control. 

### Statistical analysis

The data were analyzed with GraphPad Prism software (GraphPad Software, Inc., La Jolla, CA, USA) using one-way analysis of variance (ANOVA) for multiple comparisons, followed by Tukey's post hoc test. All numerical data are presented as the mean ± standard deviation (SD) of at least triplicate experiments. P-values of less than 0.05 were considered statistically significant.

## Results

### Effect of nargenicin A1 on the proliferation of RAW 264.7 macrophages

**Figure 1:**
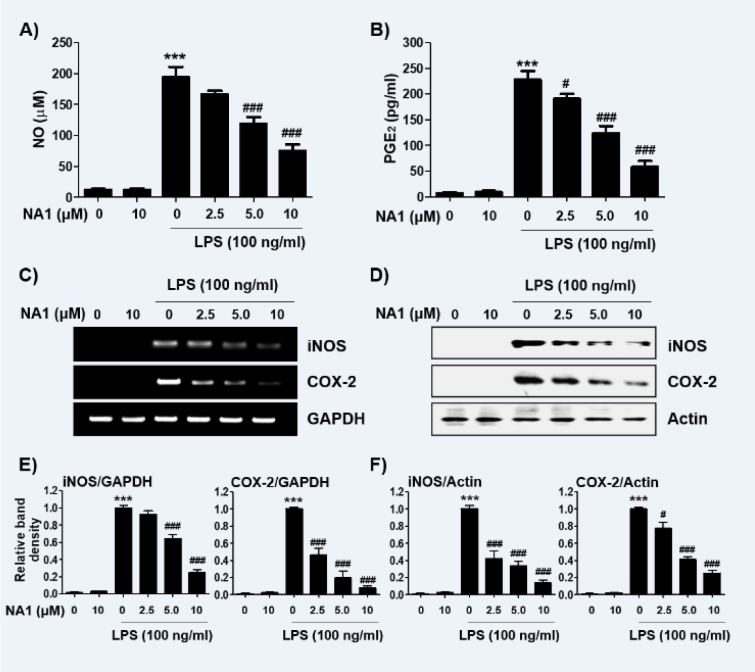
Effect of nargenicin A1 and LPS on the cell viability of RAW 264.7 macrophages. Cells were treated with various concentrations of nargenicin A1 alone for 24 h or pre-treated with or without nargenicin A1 for 1 h before 100 ng/ml LPS stimulation for 24 h. Cell viability was analyzed using the MTT assay. H_2_O_2_ was used as a positive control. Each value indicates the mean ± SD and is representative of three independent experiments. Significant differences among the groups were determined (^***^p < 0.001, *vs*. LPS-unstimulated cells).

The cytotoxic effect of nargenicin A1 on RAW 264.7 macrophages was determined by the MTT assay. As shown in Figure 1(Fig. 1), at concentrations below 10 µM, nargenicin A1 was not cytotoxic to RAW 264.7 cells. Subsequent experiments did not show any adverse effect on cell viability when 10 µM or less nargenicin A1 was administered to 100 ng/ml LPS-treated RAW 264.7 cells.

### Nargenicin A1 inhibits LPS-induced NO and PGE_2_ production in RAW 264.7 macrophages

**Figure 2:**
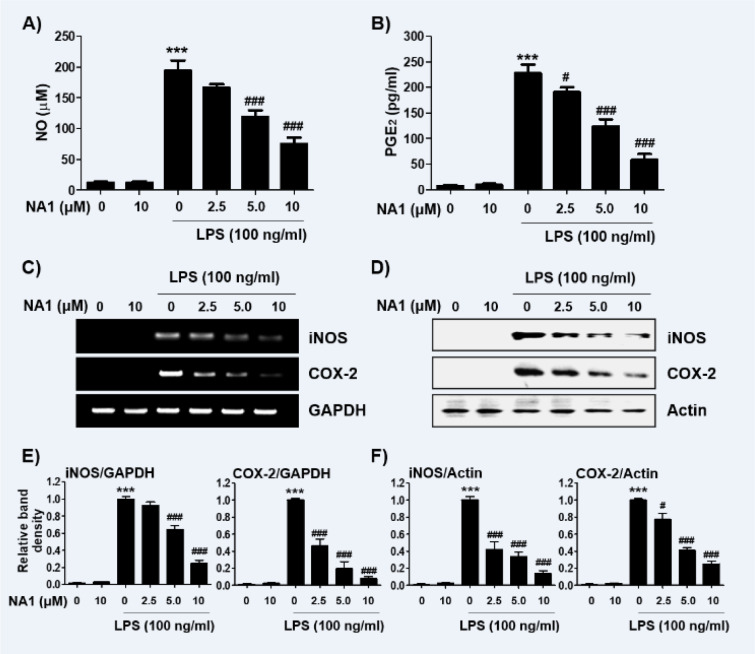
Effect of nargenicin A1 on the production and expression of pro-inflammatory mediators in LPS-stimulated RAW 264.7 macrophages. Cells were treated with the indicated concentrations of nargenicin A1 for 1 h and then stimulated with 100 ng/ml LPS for 24 h. (A) The NO concentration in the culture medium was determined by the Griess reaction. (B) The PGE_2_ concentration was determined using a commercial ELISA kit. (A and B) The absorbance was measured using a microplate reader. The error bars represent the mean ± SD of three independent experiments (^***^p < 0.001, *vs*. LPS-unstimulated cells; ^#^*p *< 0.05 and ^###^*p *< 0.001, *vs*. LPS-stimulated cells). (C and D) After treatment, total RNA and protein were extracted from the cells. The expression levels of iNOS and COX-2 mRNA (C) and proteins (D) were measured by RT-PCR and Western blot analysis, respectively. Glyceraldehyde 3-phosphate dehydrogenase (GAPDH) and actin were used as internal controls for the RT-PCR and Western blot analyses, respectively. (E and F) Bands were quantified using ImageJ and normalized to GAPDH (E) and actin (F), and the ratio was determined. Data are expressed as the mean ± SD of three independent experiments (^***^p < 0.001, *vs*. LPS-unstimulated cells; ^#^*p *< 0.05 and ^###^*p *< 0.001, *vs*. LPS-stimulated cells).

To evaluate the anti-inflammatory effects of nargenicin A1, changes in the levels of inflammatory mediators such as NO and PGE_2_ were detected. As shown in Figures 2A and B(Fig. 2), LPS treatment greatly increased the release of NO and PGE_2_ compared to untreated controls, but this increase was significantly reduced in nargenicin A1-pre-treated cells in a concentration-dependent manner. Next, we investigated whether nargenicin A1 could inhibit the expression of iNOS and COX-2 by LPS. According to the RT-PCR and Western blot results, the mRNA and protein expression of iNOS and COX-2 increased by LPS was significantly suppressed in the presence of nargenicin A1 (Figures 2C-F(Fig. 2)).

### Nargenicin A1 reduces the production and expression of LPS-induced pro-inflammatory cytokines in RAW 264.7 macrophages

**Figure 3:**
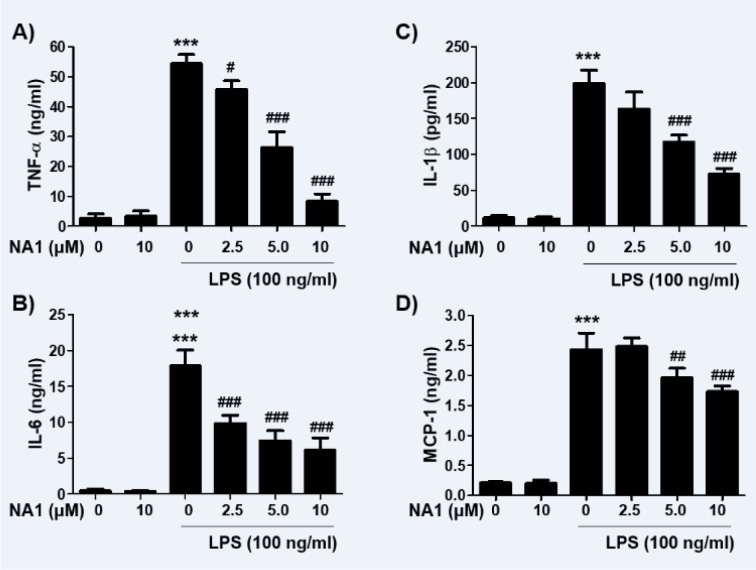
Effect of nargenicin A1 on the production of pro-inflammatory cytokines in LPS-stimulated RAW 264.7 macrophages. Cells were treated with the indicated concentrations of nargenicin A1 for 1 h and then stimulated with 100 ng/ml LPS for 24 h. The concentrations of TNF-α (A), IL-1β (B), IL-6 (C), and MCP-1 (D) in the culture medium were measured using commercial ELISA kits. The error bars represent the mean ± SD of three independent experiments (^***^p < 0.001, *vs*. LPS-unstimulated cells; ^#^*p *< 0.05, ^##^*p *< 0.01 and ^###^*p *< 0.001, *vs*. LPS-stimulated cells).

**Figure 4:**
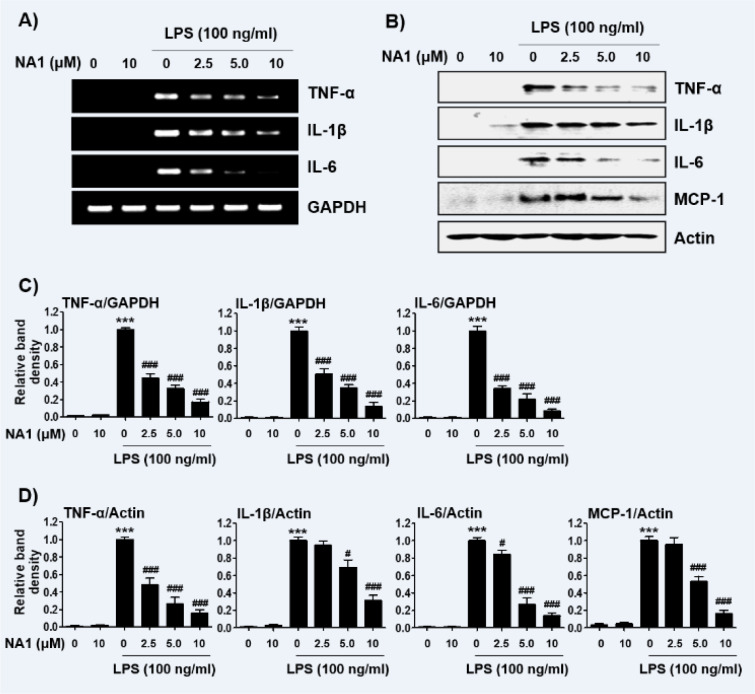
Effect of nargenicin A1 on the expression of pro-inflammatory cytokines in LPS-stimulated RAW 264.7 macrophages. Cells were treated with the indicated concentrations of nargenicin A1 for 1 h and then stimulated with 100 ng/ml LPS for 24 h. Total RNA and protein were extracted from the cells. The expression levels of pro-inflammatory cytokines mRNA (A) and proteins (B) were measured by RT-PCR and Western blot analysis, respectively. GAPDH and actin were used as internal controls for the RT-PCR and Western blot analyses, respectively. (C and D) Bands were quantified using ImageJ and normalized to GAPDH (C) and actin (D), and the ratio was determined. Data are expressed as the mean ± SD of three independent experiments (^***^p < 0.001, *vs*. LPS-unstimulated cells; ^#^*p *< 0.05 and ^###^*p *< 0.001, *vs*. LPS-stimulated cells).

Next, we investigated the effect of nargenicin A1 on the production and expression of pro-inflammatory cytokines increased by LPS treatment. Our results showed that the amount of pro-inflammatory cytokines, including TNF-α, IL-1β, IL-6, and MCP-1, after stimulation with LPS increased significantly. However, the enhanced production of these cytokines by LPS was significantly suppressed by nargenicin A1 pretreatment, and this effect was dependent upon the nargenicin A1 treatment concentration (Figure 3A-D(Fig. 3)). Subsequently, whether the inhibition of cytokine production by nargenicin A1 in LPS-treated RAW 264.7 cells was associated with the decreased expression of these genes was also investigated. As shown in Figure 4(Fig. 4), LPS treatment significantly increased the expression of these cytokine proteins, but their expression was reduced in cells pre-treated with nargenicin A1.

### Nargenicin A1 suppresses the nuclear translocation of NF-κB in LPS-stimulated RAW 264.7 macrophages 

**Figure 5:**
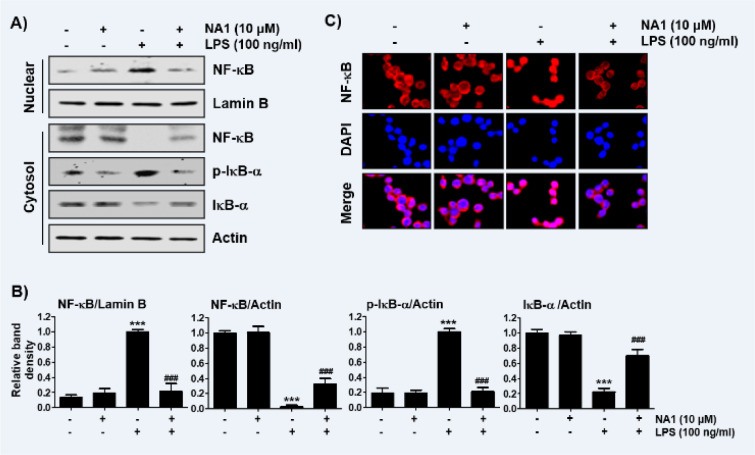
Inactivation of NF-κB signaling pathway by nargenicin A1 in LPS-stimulated RAW 264.7 macrophages. Cells were treated with 10 mM nargenicin A1 alone for 1 h or pre-treated with or without 10 mM nargenicin A1 for 1 h before 100 ng/ml LPS stimulation for 1 h. (A) For Western blot analysis, nuclear and cytosolic proteins were isolated, and the expression of NF-κB, p-IκB-a, and IκB-a was investigated. Protein loading was confirmed by the analysis of lamin B or actin expression in each protein extract. N.F., nuclear fraction; C.F., cytosolic fraction. (B) Bands were quantified using ImageJ and normalized to lamin B or actin, and the ratio was determined. Data are expressed as the mean ± SD of three independent experiments (^***^p < 0.001, *vs*. LPS-unstimulated cells; ^###^*p *< 0.001, *vs*. LPS-stimulated cells). (C) The cells were subjected to immunofluorescence staining with NF-κB p65 antibody and representative fluorescence images were acquired using a fluorescence microscope. Red fluorescence indicates the localization of NF-κB p65 and blue fluorescence by DAPI staining allows visualization of the nuclei.

It was further investigated whether nargenicin A1 inhibits the LPS-mediated activation of NF-κB because it is a key factor controlling the transcription of pro-inflammatory mediators and cytokines. As shown in Figures 5A, B(Fig. 5), when RAW 264.7 cells were stimulated with LPS, the expression of NF-κB in the nucleus was significantly increased compared with the control group. By contrast, the level of IκB-α in the cytoplasm was decreased by the treatment of LPS, which was associated with an increase in the expression of phosphorylated inhibitor IκB-α (p-IκB-α), indicating that NF-κB was activated. However, nargenicin A1 reduced the nuclear accumulation of NF-κB p65, the expression of p-IκB-α, and the degradation of IκB-α induced by LPS. Consistent with the immunoblotting results, the increase in fluorescence intensity of NF-κB p65 observed in the nuclei of LPS-treated cells was markedly decreased by pre-treatment with nargenicin A1 (Figure 5C(Fig. 5)).

### Nargenicin A1 alleviates the LPS-mediated generation of ROS in RAW 264.7 macrophages

**Figure 6:**
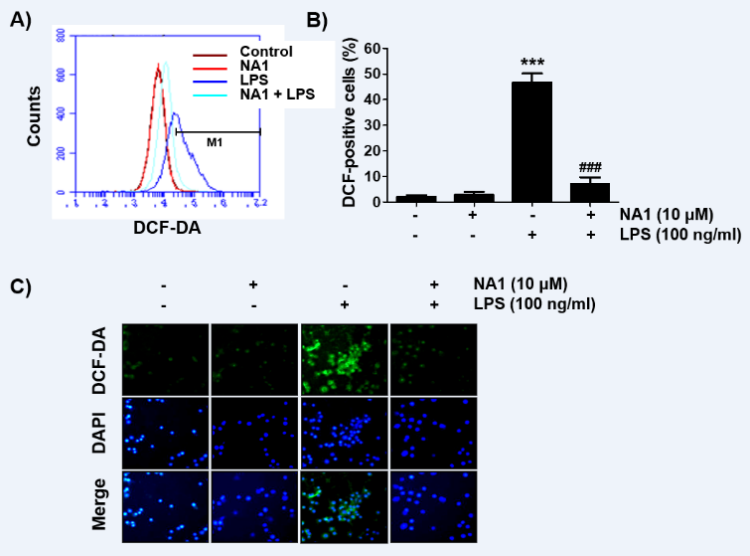
Inhibition of ROS generation by nargenicin A1 in LPS-stimulated RAW 264.7 macrophages. Cells were pre-treated with 10 mM nargenicin A1 for 1 h and then treated with 100 ng/ml LPS for 1 h. (A) The DCF-DA-stained cells were collected, and then DCF fluorescence was analyzed by flow cytometry. (B) Data are given as the mean ± SD of three independent experiments (^***^p < 0.001, *vs*. LPS-unstimulated cells; ^###^*p *< 0.001, *vs*. LPS-stimulated cells). (C) ROS generation was also detected by a fluorescence microscope and representative fluorescence micrographs depicting ROS generation are presented. Green fluorescence indicates the intensity of ROS generation and blue fluorescence by DAPI staining allows visualization of the nuclei.

Since oxidative stress also plays an important role in the activation of macrophages and inducing inflammatory responses, we investigated whether nargenicin A1 suppresses LPS-induced oxidative stress using the DCF-DA probe. The flow cytometry results showed that the levels of intracellular ROS contents increased with the stimulation of LPS (Figures 6A, B(Fig. 6)). However, the increase in ROS content in RAW 264.7 cells treated with LPS was dramatically reduced by the addition of nargenicin A1. Consistent with the results from the flow cytometry, the increase in the fluorescence intensity of DCF-DA observed in the cells treated with LPS was weakened by pretreatment of nargenicin A1 (Figure 6C(Fig. 6)).

### Nargenicin A1 weakens the production of NO and ROS in LPS-treated zebrafish larvae

**Figure 7:**
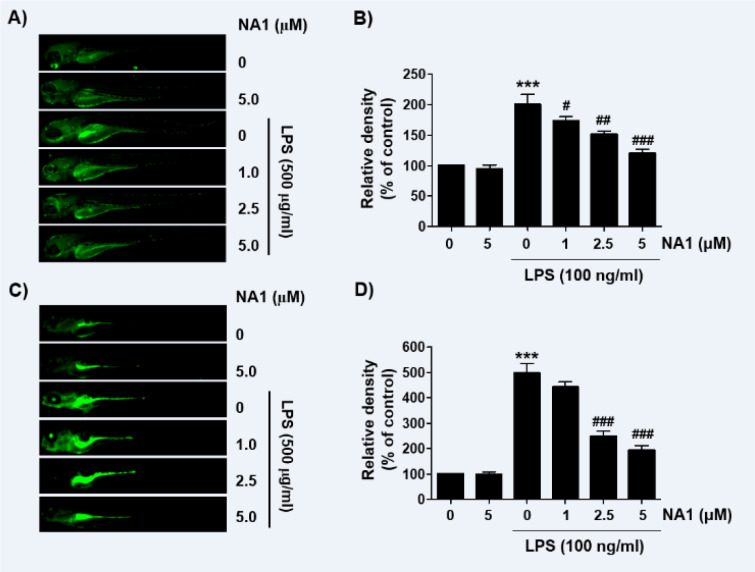
Inhibition of LPS-induced NO and ROS generation by nargenicin A1 in zebrafish larvae. Zebrafish at 3 dpf were microinjected with 2 nL of 0.5 mg/ml LPS and placed in E3 media containing the indicated concentrations of nargenicin A1 for 24 h. The larvae were incubated with 5 µM DAF-FM-DA (A and B) or 20 µM DCF-DA (C and D) for NO and ROS detection and visualized using the CELENA® S Digital Imaging System. (B and D) Relative fluorescence intensities were calculated and expressed compared to the untreated control. Each value represents the mean ± SD and represents 20 fishes for each group. Significant differences among the groups were determined (^***^p < 0.001, *vs*. LPS-unstimulated larvae; ^#^*p *< 0.05, ^##^*p *< 0.01 and ^###^*p *< 0.001, *vs*. LPS-stimulated larvae).

As nargenicin A1 downregulates inflammatory and oxidative responses in RAW 264.7 cells, we wondered if nargenicin A1 exhibited similar effects in the *in vivo* model and demonstrated using a zebrafish model. According to the present results of DAF-FM-DA staining, LPS microinjection significantly increased NO generation. However, in the presence of nargenicin A1, the LPS-induced NO generation gradually decreased in a concentration-dependent manner (Figures 7A and B(Fig. 7)). In addition, we confirmed by DCF-DA staining that the increased ROS production in LPS-microinjected zebrafish larvae was concentration-dependently abrogated in the presence of nargenicin A1 (Figures 7C and D(Fig. 7)).

## Discussion

The activation of macrophages is essential to defending against inflammation caused by external stimuli, but excessive inflammatory reactions contribute to the onset and progression of inflammation-related diseases (Hernandez et al., 2019[16]; Doyle and O'Neill, 2006[11]). In addition, ROS generated from overactive macrophages induce oxidative damage and can serve as a secondary messenger that further amplifies the inflammatory cascade, and inflammation-inducing factors are also involved in the production of ROS (Liu et al., 2018[22]; Mills and O'Neill 2016[25]; Lee and Yang, 2012[21]). 

In this study, to evaluate the anti-inflammatory efficacy of nargenicin A1, we first investigated its effect on the production of NO and PGE_2_. Among them, NO, which is synthesized from L-arginine by NO synthase, plays a critical role in normal physiological conditions such as neurotransmission, vasodilation, and immune defense. However, excessive NO formation due to increased iNOS expression promotes the inflammatory response and increases oxidative stress and tissue damage (Yao and Narumiya, 2019[39]; Saini and Singh, 2019[31]). COX enzymes catalyze the conversion of arachidonic acid to prostaglandins, including PGE_2_, a group of hormone-like substances that participate in various body functions (Aleem and Tohid, 2018[2]; Soufli et al., 2016[35]). However, excessive PGE_2_ production, promoted by the increased activity of COX-2 following various inflammatory stimuli, plays an important role as an inflammatory mediator (Yao and Narumiya, 2019[39]; Saini and Singh, 2019[31]). Therefore, inhibitors of the excessive production of these inflammatory mediators can be regarded as therapeutic agents against inflammation-related diseases. Our data indicated that the up-graduated secretion of NO and PGE_2_ by LPS in RAW 264.7 macrophages was progressively inhibited at increasing concentrations of nargenicin A1, which was associated with inhibition of the expression of iNOS and COX-2 mRNA and protein. These results indicated that the anti-inflammatory effect of nargenicin A1 was at least due to the reduced expression of iNOS and COX-2, which are required for NO and PGE_2_ production.

During the inflammatory response, macrophages secrete multiple pro-inflammatory cytokines through various signaling pathways (Hu et al., 2020[17]; Zhang et al., 2020[41]; Wang et al., 2017[37]). All of these are essential components for the initiation and improvement of the inflammatory response, and their expression is also increased by the LPS stimulation of macrophages (Aleem and Tohid, 2018[2]; Wyns et al., 2015[38]). Moreover, they can accelerate the inflammatory response by activating or increasing the expression of pro-inflammatory mediators as well as other pro-inflammatory cytokines (Zhang et al., 2020[41]; Soufli et al., 2016[35]). Although the expression of pro-inflammatory cytokines is tightly controlled by transcriptional and post-transcriptional mechanisms, they may have similar or even the same functional activity (Hu et al., 2020[17]; Popa et al., 2007[28]). Therefore, the level of pro-inflammatory cytokines has been applied as an indicator to evaluate anti-inflammatory efficacy in macrophages. In this study, we found that nargenicin A1 reduced the production of TNF-α, IL-1β, and IL-6 in LPS-stimulated RAW 264.7 macrophages by suppressing their expression. 

As mentioned in many previous studies, NF-κB plays a critical role in the control of inducible anti-inflammatory enzymes and cytokines in LPS-activated macrophages (Hernandez et al., 2019[16]; Doyle and O'Neill, 2006[11]). Typically, NF-κB forms a complex with the inhibitory subunit IκB-α and remains inactive in the cytoplasm. When IκB-α is phosphorylated through the upstream signaling systems by inflammatory stimuli such as LPS and degraded by the ubiquitin-proteasome system, NF-κB migrates to the nucleus, triggering transcriptional activation of inflammation-inducing genes and catabolic enzymes (Schuliga, 2015[32]; Rigoglou and Papavassiliou, 2013[29]). Therefore, the efficacy of nargenicin A1 on LPS-induced NF-κB activation was further evaluated because blocking the activity of NF-κB could be effective in treating inflammation. The current data demonstrated that the nuclear translocation of NF-κB, and the degradation and phosphorylation of IκB-α were increased in LPS-treated RAW 264.7 macrophages, but nargenicin A1 greatly prevented the nuclear translocation of NF-κB and the degradation and phosphorylation of IκB-α. Therefore, the inhibitory effect of nargenicin A1 on the increased expression of pro-inflammatory enzymes and cytokines in LPS-stimulated RAW 264.7 cells is due to blocking of the translocation of NF-κB from the cytoplasm to nucleus, and these results are in good agreement with the anti-inflammatory mechanism of this compound found in LPS-stimulated microglial cells (Yoo et al., 2009[40]).

Meanwhile, endogenous free radicals such as ROS play a play important role in host defense. However, excess ROS can cause oxidative damage to cellular macromolecules, and has been shown to play an important role in initiating and promoting inflammation-related diseases by upregulating the production of inflammatory mediators and cytokines (Liu et al., 2018[22]; Bjørn and Hasselbalch, 2015[4]). ROS also contribute to the activation of macrophages, and ROS generation is enhanced in overactive macrophages (Liu et al., 2018[22]; Mills and O'Neill 2016[25]). According to our results, nargenicin A1 strongly inhibited ROS generation in LPS-treated RAW 264.7 macrophages. Although the antioxidant potential of nargenicin A1 has been reported in our previous study (Park et al., 2019[26]), the results of this study support the use of nargenicin A1 as an antioxidant for the management of oxidative stress associated with inflammatory responses. Since *in vivo* experiments can provide a better understanding of efficacy assessment at the organism level, the anti-inflammatory and antioxidant potential of nargenicin A1 identified in macrophages was further confirmed in the zebrafish model. The present results showed the ability of nargenicin A1 to inhibit inflammatory and oxidative reactions in the LPS-microinjected zebrafish larvae model by reducing NO and ROS generation. Although these results support our *in vitro* results, additional mechanistic studies are needed to interpret the mechanisms involved in the anti-inflammatory and antioxidant efficacy of nargenicin A1 in *in viv*o model. In addition, further studies are needed to determine the role of other cellular signaling pathways that may be involved in the anti-inflammatory activity of nargenicin A1 other than the NF-κB signaling pathway, and to determine the direct relationship with NF-κB signaling.

## Conclusion

In summary, the current study showed that nargenicin A1 could inhibit LPS-induced inflammatory and oxidative responses in RAW 264.7 macrophages and zebrafish larvae, which was evidenced by the reduced release of pro-inflammatory factors and the inhibition of ROS accumulation. Nargenicin A1 also suppressed the nuclear translocation of NF-κB and degradation of IκB-α in LPS-treated RAW 264.7 macrophages, indicating that the anti-inflammatory and antioxidant effects of nargenicin A1 on LPS stimulation are, at least, related to the inactivation of the NF-κB signaling pathway in RAW 264.7 macrophages. Therefore, it can be concluded that nargenicin A1 has potential anti-inflammatory and antioxidant activities that require extensive research for future clinical therapeutic applications.

## Conflict of interest

The authors declare that there is no conflict of interest. 

## Acknowledgement

This research was a part of the project titled “Omics based on fishery disease control technology development and industrialization” (grant no. 20150242), funded by the Ministry of Oceans and Fisheries, Republic of Korea.
