# Nargenicin A1 attenuates lipopolysaccharide-induced inflammatory and oxidative response by blocking the NF-κB signaling pathway

**DOI:** 10.17179/excli2025-8358

**Published:** 2025-03-24

**Authors:** Da Hye Kwon, Gi-Young Kim, Hee-Jae Cha, Suhkmann Kim, Heui-Soo Kim, Hye-Jin Hwang, Yung Hyun Choi

**Affiliations:** 1 Anti‐Aging Research Center, Dong‐eui University, Busan, Republic of Korea; 2 Department of Biochemistry, Dong‐eui University College of Korean Medicine, Busan, Republic of Korea; 3 Department of Marine Life Science, School of Marine Biomedical Sciences, Jeju National University, Jeju, Republic of Korea; 4 Department of Parasitology and Genetics, College of Medicine, Kosin University, Busan, Republic of Korea; 5 Department of Chemistry, College of Natural Sciences, Pusan National University, Busan, Republic of Korea; 6 Department of Biological Sciences, College of Natural Sciences, Pusan National University, Busan, Republic of Korea; 7 Department of Food and Nutrition, College of Nursing, Healthcare Sciences & Human Ecology, Dong-eui University, Busan, Republic of Korea

## Corrigendum

Corrigendum to original article

**Nargenicin A1 attenuates lipopolysaccharide-induced inflammatory and oxidative response by blocking the NF-κB signaling pathway**

EXCLi Journal 2021;20:968-982

968

**Correction of duplicated figures in http://dx.doi.org/10.17179/excli2021-3506**

In the article "Nargenicin A1 attenuates lipopolysaccharide-induced inflammatory and oxidative response by blocking the NF-κB signaling pathway" published in EXCLI Journal (EXCLI J. 2021 May 28;20:968-982. doi: 10.17179/excli2021-3506), a duplication error was identified in Figures 1 and 2. Both figures, though described differently in their figure legends, were found to be identical. Figure 1 should be:(Fig. 1)

Figure 2 is correct.

Editorial Office/EXCLI Journal on behalf of the corresponding author

This is an Open Access article distributed under the terms of the Creative Commons Attribution License 

http://creativecommons.org/licenses/by/4.0/.

## Figures and Tables

**Figure 1:**
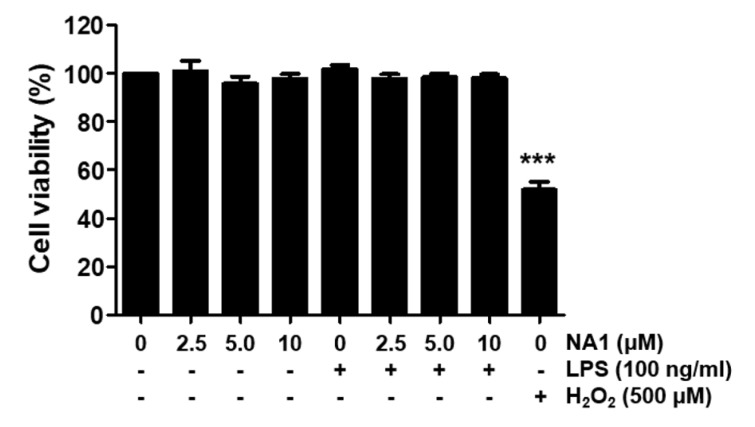
Effect of nargenicin A1 and LPS on the cell viability of RAW 264.7 macrophages. Cells were treated with various concentrations of nargenicin A1 alone for 24 h or pre-treated with or without nargenicin A1 for 1 h before 100 ng/ml LPS stimulation for 24 h. Cell viability was analyzed using the MTT assay. H_2_O_2_ was used as a positive control. Each value indicates the mean ± SD and is representative of three independent experiments. Significant differences among the groups were determined (^***^p < 0.001, *vs*. LPS-unstimulated cells).

